# Sensory Profile and Acceptability of HydroSOStainable Almonds

**DOI:** 10.3390/foods8020064

**Published:** 2019-02-12

**Authors:** Leontina Lipan, Marina Cano-Lamadrid, Mireia Corell, Esther Sendra, Francisca Hernández, Laura Stan, Dan Cristian Vodnar, Laura Vázquez-Araújo, Ángel A. Carbonell-Barrachina

**Affiliations:** 1 Department of Agro-Food Technology, Escuela Politécnica Superior de Orihuela, Universidad Miguel Hernández de Elche, Carretera de Beniel, km 3.2, 03312 Orihuela, Alicante, Spain; leontina.lipan@goumh.umh.es (L.L.); marina.cano.umh@gmail.com (M.C.-L.); esther.sendra@umh.es (E.S.); 2 Departamento de Ciencias Agroforestales, ETSIA, Universidad de Sevilla, Carretera de Utrera, km 1, 41013 Sevilla, Spain; mcorell@us.es; 3 Unidad Asociada al CSIC de Uso sostenible del suelo y el agua en la agricultura (US-IRNAS), Crta de Utrera km 1, 41013 Sevilla, Spain; 4 Department of Plant Science and Microbiology, Escuela Politécnica Superior de Orihuela, Universidad Miguel Hernández de Elche, Carretera de Beniel, km 3.2, 03312 Orihuela, Alicante, Spain; francisca.hernandez@umh.es; 5 Faculty of Food Science and Technology, University of Agricultural Sciences and Veterinary Medicine Cluj-Napoca, 400372 Cluj-Napoca, 3-5 Manastur Street, Romania; laurastan@usamvcluj.ro (L.S.); dan.vodnar@usamvcluj.ro (D.C.V.); 6 Technological Center in Gastronomy, BCC Innovation (Basque Culinary Center Research and Innovation Center), Juan Avelino Barriola 101, 20009 Donostia-San Sebastián, Gipuzkoa, Spain; lvazquez@bculinary.com; 7 Basque Culinary Center, Mondragon Unibersitatea, Juan Avelino Barriola 101, 20009 Donostia-San Sebastián, Gipuzkoa, Spain

**Keywords:** cross-cultural affective test, descriptive sensory analysis, hydroSOStainable products, *Prunus dulcis*, willingness to pay

## Abstract

Fresh water availability is considered highly risky because it is a finite resource, and a deficiency in water leads to numerous economic and environmental issues. Agriculture is one of the main consumers of fresh water in practices such as irrigation and fertilization. In this context, the main objectives of this study were (i) to determine the descriptive sensory profiles of four almond types grown using different irrigation strategies and (ii) to study their acceptance in a cross-cultural study (Romania and Spain). Consumers’ willingness to pay for hydroSOS almonds was also evaluated. The four irrigation strategies evaluated were a control sample, two samples grown under regulated deficit irrigation strategies (RDI), and a sample grown under a sustained deficit irrigation strategy (SDI). The main conclusion was that neither descriptive nor affective sensory results showed significant differences among treatments. These findings should encourage farmers to reduce their water usage by demonstrating that sensory quality was not significantly affected by any of the studied treatments, compared to the control. Regarding willingness to pay, both Spanish and Romanian consumers were willing to pay a higher price for the hydroSOS almonds.

## 1. Introduction

Fresh water is a finite resource, and uncertainty regarding the remaining level of water for future generations has led the world to seek sustainability as a compulsory issue for future economic development and healthy ecosystems [1,2]. The World Economic Forum (WEF) placed water scarcity as the main global risk of the economy regarding impact, because a shortage of water means a stoppage of factories and food production, leading to the decline of the global economy [2]. The population growth drives to an augmentation in intensive food production that alters the environment due to greenhouse gas emissions, soil deterioration, and water stress [3]. 

Agriculture is one of the biggest consumers of fresh water, mainly due to the large volume necessary for irrigation (70–80% of the total) [1,4]. Opinions about irrigation in agriculture are divided about whether irrigation is necessary or not. Some believe water irrigation is required to produce enough food in the future due to world population growth, while others find irrigation agriculture wasteful because it creates ”water-guzzling crops” [4]. For this reason, agriculture, particularly in the Mediterranean (semi-arid) region, must evaluate water use sustainability by implementing plans and irrigation strategies capable of reducing water irrigation but maintaining the quality of products [5]. 

Almonds are the major tree nut crop in the Mediterranean basin, which is defined by low rainfall and elevated evaporated demand during the almond growing cycle [6]. Although it is considered a drought-resistant crop, the almond tree (*Prunus dulcis*) needs irrigation to produce yield and profitability [6,7]. Numerous studies in fruits, such as almonds, olives, pistachio, apples, and grapes, have proven that fruit quality could be increased by controlling and reducing the amount of water irrigation [8,9,10,11,12,13,14]. Therefore, the development of deficit irrigation strategies (DI), such as regulated and sustained deficit irrigation, might be useful to increase the water productivity maintaining fruit quality.

Deficit irrigation strategies refer to the application of water below the crop evapotranspiration (ET: the combination between the evaporation losses from the soil and transpiration losses from the crop) requirements [4]. Regulated deficit irrigation (RDI) was developed to supervise vegetative vigor and consists of applying limited water during certain stages (in which plant is less sensitive to water stress) of the growing season. In the almond crop, the most recommended and less sensitive phenological period to apply water stress is the stage IV, which is contemporaneous with kernel filling and happens during the summer months of highest evaporative demand [5,6]. On the other hand, sustained deficit irrigation (SDI) is a strategy in which a uniform and reduced amount of water is applied to crops during all growing cycles, creating a progressive stress in plants throughout the season [6]. In this strategy, stress is produced by not entirely refilling the root zone when irrigated [7].

Sensory analysis techniques are essential to establish the quality of a product and to understand consumer preferences. Descriptive sensory analysis consists of detection and description, not only of quantitative but also qualitative sensory attributes of products. These attributes are of utmost importance to define a product, including its appearance, aroma, flavor, and texture [15]. On the other hand, affective tests are used to evaluate consumer preferences or acceptance responses to a product [15]. 

Society now expects the incorporation of an environmental sustainability plan [16]. In this context, consumers play an essential role because they demand and choose food products with specific characteristics, and nowadays they are concerned, not only about healthy diets but about environmental protection [3]. This has led to the development of the “environmentalism” phenomenon, which mostly relies on government agencies and non-governmental organizations caring about ecological issues [17]. These phenomena have made the consumer more conscious and interested in healthy, safe, and environmentally friendly food; consequently, the consumer has a greater willingness to pay for eco-friendly and hydroSOStainable (hydroSOS) products [17,18]. 

Under these circumstances, the aim of this study was (i) to determine the descriptive sensory profiles of four different almond types grown using different irrigation strategies (including hydroSOS samples) and (ii) to study their acceptance and consumers’ willingness to pay in a cross-cultural study in Romania and Spain. Understanding consumers’ preferences and their willingness to pay for hydroSOS almonds is vital for almond growers. 

## 2. Materials and Methods

### 2.1. Irrigation Treatments

The almond cultivar used in the present study was “Vairo” and was grown on the commercial farm “La Florida” located in Dos Hermanas (Seville, Spain). The following four irrigation treatments were evaluated:

▪ **T1** was full irrigation treatment using 433 ± 26 mm of applied water throughout the season with a stress integral of SI = 54.2. Trees were irrigated to assure the estimated crop ET, and thus represented the control.
▪ **T2** were trees under regulated deficit irrigation (RDI) at optimum level (148 ± 24 mm; SI = 91.7). For irrigation scheduling, midday stem water potential (SWP) and maximum daily shrinkage (MDS) measurements were done. Then, in stage IV (kernel filling) of the almond growing cycle, the trees were irrigated when SWP was lower than −1.5 MPa or when MDS signal was above 1.75. The rest of the stages were irrigated to the SWP proposed by McCutchan and Shackel (1992) or MDS equal 1 [19].
▪ **T3** trees were also irrigated under regulated deficit irrigation but in more severe conditions (103 ± 13 mm; SI = 94.9). Thus, the stage IV trees were irrigated when SWP was lower than −2 MPa or MSD signal above 2.75, and similar conditions as previously described for T2 were applied for the rest of the period.
▪ **T4** trees were irrigated under sustained deficit irrigation (SDI) conditions (114 ± 13 mm; SI = 74.7). Water was applied gradually throughout the growing season.

In order to determine the accumulative effect of water deficit, water stress integral was calculated by using the following equation:

(1) $$\mathrm{SI}=|\sum^{\;}\left(\min\;\Psi_{\mathrm{stem}}-\left(-0.2\right)\right)\;\times \;n$$

In this expression, SI was the stress integral, min Ψ_stem_ was the average of minimum SWP, and *n* represented the day numbers interval. 

The field study was conducted during 2017; in August, almonds were harvested, dried (below 5% moisture content), and delivered to University Miguel Hernández of Elche (Spain) facilities, where descriptive and affective studies were carried out. Almonds were also sent to University of Agricultural Sciences and Veterinary Medicine of Cluj-Napoca (Romania) to perform affective studies with Romanian consumers. Around 1.5 kg of almond kernels was needed for each treatment.

### 2.2. Descriptive Sensory Analysis

A trained panel with 10 highly trained panelists from the Food Quality and Safety Group (Miguel Hernández University of Elche, Orihuela, Alicante, Spain) conducted the descriptive analysis. Each panelist had more than 600 hours of experience with different types of food products. Although the panel had a vast experience in tasting almond and turrón (traditional Spanish dessert made basically of toasted almonds and honey), they had four orientation sessions for the almond tasting, where the panelists decided the final list of descriptors and reference products for each attribute. The reference and modified lexicon were the ones developed by Vázquez-Araújo et al. [20]. Table 1 shows the reference products used by the panelists for flavor and texture characterization. The almond color scale was developed using instrumental color measurements carried out with a Minolta Colorimeter CR-300 (Minolta, Osaka, Japan) in 400 almonds. The minimum, mean, and maximum values from instrumental color intensities were later converted into pantones with an online program Nix Color Sensor [21] and presented to the panelists as references. The ∆*E* shows the degree of total color change [22] and was calculated as,

∆*E* = [(*L* − L*)^2^ + (*a* − a*)^2^ + (*b* − b*)^2^]^0.5^ (2)

Almond roughness was visually measured and refers to the number of hills and valleys perceived by the human eye on the almond surface [23]. Almond size scale was prepared from the one used by Regulating Council of the Protected Geographical Indications of *Jijona and Turrón de Alicante* (RCPGIJTA) [24]. 

The texture attributes (Table 1) products were also analyzed using instrumental texture measurements. A texture analyzer (Stable Micro Systems, model TA-XT2i, Godalming, UK) was employed using a 30 kg load cell and a Volodkevich Bite Jaw HDP/VB probe (trigger was set at 15 g, test speed was 1 mm s^−1^ over a specified distance of 3 mm).

After the orientation session, each panelist received four samples corresponding to the different irrigation treatments, and three evaluations per sample were done. The samples were served in odor-free 30 mL covered plastic cup and randomly coded with three digits. Water and unsalted crackers were also provided in order to clean the palates among samples. The descriptive test was carried out in a special tasting room with individual booths (controlled temperature of 21 ± 1 °C and combined natural/artificial light), and ballot charts were used to collect panelists’ evaluations. The samples were presented according to a randomized block design to avoid biases. A 0 to 10 numerical scale was used by the panelists to quantify the intensity of the almond attributes, where 0 represents none/no intensity and 10 extremely strong with a 0.5 increment. 

### 2.3. Affective Sensory Analysis

Affective sensory analysis was carried out with 100 recruited consumers from Spain (S) and 100 from Romania (R), with a gender ratio of 50:50 in Spain and 60:50 women:men in Romania. The consumers’ age range was 18–25 (S = 33%; R = 45%), 26–35 (S = 29%; R = 30%), 36–45 (S = 10%; R = 15%), and 45–60 (S = 29%; R = 10%). The recruitment process was conducted via e-mail and fliers. Demographic questions regarding gender, age, nut consumption frequency, allergies, intolerances, or diet restriction were also included in the questionnaire. Spanish to Romanian back-translation procedure was conducted to avoid major misunderstandings during the evaluation. All samples were served, and labeled with three digit codes, in the same manner as with the recipients as described above. Consumers were asked for global satisfaction degree using a 9-point hedonic scale (1 = dislike extremely and 9 = like extremely) for scoring and about attributes intensity using Just About Right (JAR) questions. Consumers were also asked to rank samples according to their preference and to check the reasons why they choose that sample as the best (due to the color, flavor sweetness, crunchiness, etc.) by using a question type Check All That Apply (CATA). Consumer interest in the label information (sustainable, bio, healthy, natural, product of Spain/Romania, etc.) using CATA question type was also analyzed. As described in the descriptive section, the affective tests were also carried out in special tasting rooms with individual booths and according to a randomized block design.

### 2.4. Consumer Willingness to Pay

The willingness to pay was carried out with 100 consumers form Spain and 100 consumers from Romania. Both Spanish and Romanian consumers were first given information about what the hydroSOStainable concept means, and later they were asked for their willingness to pay for hydroSOS almonds compared to the conventional ones. It was decided to inform consumers about hydrosustainability, because it was a relatively new concept and because previous studies have demonstrated that consumers need enough knowledge and access to precise information to prevent the receiving of fake feedback [3]. Without this previous basic information about the hydroSOStainable concept, consumers’ responses and resulting conclusions with regard to hydroSOS almonds would be deeply speculative [25]. Later, they were given a price for conventional almonds of 2.60 €/200 g (the normal price for the Mercadona almonds; Mercadona is one of the most popular food supermarkets in the Mediterranean area of Spain) and the options: ≤€2.60, €3.10, €3.60, and >€3.60.

### 2.5. Statistical Analysis

Statistical analyses were performed by subjecting the data to two or three-way analysis of variance (ANOVA) and then to Tukey’s multiple range test. A three-way ANOVA (factor 1: irrigation treatment; factor 2: session; and, factor 3: panelist) was carried out to demonstrate the panel consistency in the descriptive sensory analysis data, while two-way ANOVA (factor 1: irrigation treatment, and factor 2: country) was used for the affective sensory data [26]. Statistically significant differences were considered when *p* < 0.05, and were performed using XLSTAT Premium 2016 (Addinsoft, New York, NY, USA) and Statgraphics Plus (Version 3.1, Statistical Graphics Corp., Rockville, MA, USA). 

Penalty analysis was also carried out to supply information about the possible improvement of samples, and for these analyses, JAR data were used [26]. Mean drops (penalties) versus the percentage of the consumers (providing each response in the mean drop plot) were graphically represented. 

## 3. Results and Discussion

### 3.1. Descriptive Sensory Analysis

The descriptive sensory analysis was performed to evaluate whether significant differences among treatments were found. The descriptive results showed no statistically significant differences for session and panelist, and their two-way interaction demonstrated proper performance of the panel and the lack of effects of the parameters panelist and replication. Thus, only the effect of the parameter “irrigation treatment” is presented and discussed in the manuscript. 

Table 2 shows the effect of the studied irrigation treatments on the main sensory descriptors of control and hydroSOS almonds. No significant differences were observed for 12 out of the 17 attributes used to describe the quality of almonds, while statistically significant differences were found for color, size, roughness, sweetness, and hardness. 

Panelists found T2 samples having more intense color. This was supported by the instrumental color data, which showed significantly higher values for the a* coordinate (T1 = 16.7 b; T2 = 17.4 a; T3 = 17.1 ab; T4 = 17.3 ab). Although the differences for *AE* color were below two units, and differences are difficult to perceive with the human eye [27], the highest values in the a* (green-red coordinate) indicated that T2 almonds were more reddish. However, other authors obtained no statistically significant differences for this parameter in pistachio and olives [8,10,28]. However, other authors showed that total color increased for apricots and peaches under RDI [29,30]. 

With respect to the size attribute (Table 2), no significant differences were observed in instrumental size (mm) among treatments (T1 = 16.3; T2 = 16.2; T3 = 16.2; T4 = 16.2), but slightly significant differences were observed in descriptive analysis for T1 and T2, compared to a second group, T3 and T4. The results were partially similar to previous studies about hydroSOS pistachios, in which it was observed that there were no significant differences either for sensory or for instrumental size [10]. It is a general working hypothesis that deficit irrigation can lead to reduced yield, but the fruits produced will be of higher size. The problem of working with fruits, such as almonds, is that the heterogeneity of the fruits is so high that in some cases and parameters/attributes it can mask real differences due to the applied treatments. This is one reason, among others, for this hypothesis not being confirmed by real data. However, current field experiments are being repeated during three years to have more realistic and reliable data, but preliminary data is needed to be able to implement improvements in the experiment design and reach partial goals and objectives.

Although the roughness (Table 2) of T2 and T4 recorded the highest values, they were within the optimal values, which meant that water stress was correctly applied. Applying water stress at the wrong growing stages, for instance, stage III, will lead to very rough kernels, which are indicative that the water stress also reached the fruit and its turgor and moisture content were drastically limited [31]. 

An important finding was that T2 and T3 were the sweetest samples. An increment in sweetness was demonstrated in “Mollar de Elche” pomegranate cultivar growth under deficit irrigation conditions [32]. Sweetness is a key attribute in the sensory quality of almonds, and it is expected that increased sweetness intensity will be favorable for consumer satisfaction [33]. Table 2 shows texture results, and only significant differences were found for hardness; T1 almonds were slightly softer than those from the rest of treatments. However, no differences were found for the instrumental hardness (T1 = 73.8 N; T = 73.8 N; T3 = 72.8 N; T4 = 72.2 N). Other authors also showed higher values for both sensory and instrumental texture for DI samples in studies about pistachios and olives samples [8,10]. 

### 3.2. Affective Sensory Analysis

Table 3 showed that the overall and attribute specific satisfaction degree of both Spanish and Romanian consumers were not statistically affected by the irrigation strategies under evaluation, with the exception of T4 almonds causing a slightly higher satisfaction. In general, Romanians tend to score higher than Spanish consumers because of the fact that they are less used to consuming this nut. Other authors, in studies about olives under deficit irrigation conditions, also reported no significant differences among the treatments for affective sensory evaluation [9]. On the contrary, there are also plenty of works on olives, pistachio, peaches, and grapes, in which higher consumer acceptance for samples produced by deficit irrigation strategies, such as RDI (moderate level), were observed [8,10,34,35]. 

**Figure 1:**
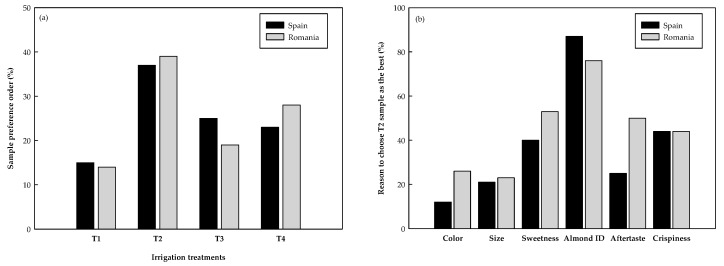
Purchase intent of Spanish and Romanian consumers regarding the studied almonds (**a**), and their reason to choose T2 almonds as the favorite ones (**b**).

Figure 1 shows the sample preference order of Spanish (S) and Romanian consumers (R), and the main attributes controlling their preference. Sample T2 was chosen by consumers from both countries as the most liked sample, while T1 almonds were the least like ones (Figure 1a). Spanish consumers scored T4 higher than T3, while the contrary was observed from the Romanian consumers. T2 almonds were chosen as the best ones mainly due to their almond flavor, sweetness, and crispiness (Figure 1b), showing that sweetness was important in consumers’ satisfaction, as hypothesized before in this study. 

Besides, sweetness (S = 29%; R = 64%), flavor (S = 77%; R = 65%), texture (S = 44%; R = 16%), and price (S = 44%; R = 60%) were the most checked parameters in the CATA questionnaire used when consumers were asked about their buying drivers. The most important word in a product label for 63% of the Spanish consumers was “product of Spain”, followed by “healthy” (52%) and “natural” (48%), while Romanian consumers were more interested in “natural” (70%), “healthy” (67%), and “ecological” (31%). For both nationalities, the words “natural” and “healthy” seemed to play a key role in their buy decisions. Noguera et al. [18] also found the word “product of Spain” as the most important attribute for Spanish consumers concerning hydroSOS pistachio along with other expressions such as “rich in antioxidants” and “crunchy”. The findings were associated with the consumer recognition of national products, health concerns, and composition. Regarding the word “sustainable”, 44% of Spanish consumers and 29% of Romanians were interested in this word when purchasing a product. Although it is a relatively new concept [36], other authors also showed great interest of Spanish consumers for this word and concept but when studying other products, pistachios [18].

**Figure 2:**
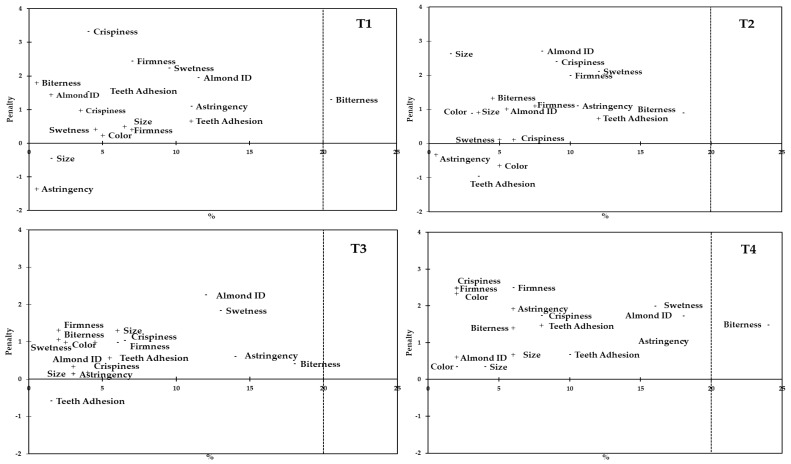
Penalty analysis of attributes intensities assessed by consumers (sample code indicated on the top right of each figure; “too low intensity” is indicated by the symbol “−“, and “too high intensity” is indicated by the symbol “+”).

Penalty analysis, a very popular method in the food industry sector to help one interpret data from JAR questions, was conducted to understand the relationship between consumers’ overall liking and the attribute intensity scores of the JAR questions [37,38]. The attributes with a large penalty and a high percentage of consumers, which were placed in the upper right quadrant of the plots, provided information about the most critical diagnostic issues of the product. On the other hand, the preferred attributes were usually located at the lower left quadrant of the plots [26]. The proportion of consumer’s opinion plots and the mean penalty is shown in Figure 2 for all four treatments under study. All attributes having a negative impact on the sample liking, for at least 20% of the consumers and producing a drop of at least 1 point for liking, are the ones that might need to be improved. Bitterness was the only parameter susceptible to be improved, and consumers from both Spain and Romania agreed that this was especially true for T1 and T4 almonds. 

### 3.3. Consumer Willingness to Pay

The Spanish and Romanian consumers were classified according to their willingness to pay for a bag of hydroSOS almonds compared to a bag of conventional almonds: (i) S = 23% and R = 31% were willing to pay less or the same price; (ii) S = 60% and R = 16% wanted to pay 0.50 € more; (iii) S = 13% and R = 24% wanted to pay 1.00 € more; and, finally, (iv) S = 4% and R = 29% wanted to pay more than 1.00 €. These findings agreed with Noguera et al. [18], who reported that Spanish consumers were also willing to pay an extra amount of money for hydroSOS pistachios [18]. 

Considering the almond Spanish production of ~ 190.000 t, the price received by the farmers for conventional shelled almonds was ~ 4.85 € kg^−1^ [39], and according to the previous data, an extra value of ~2 € kg^−1^ hydroSOS almonds can be expected. The possible economic increase by using deficit irrigation strategies could be ~40% with respect to conventional almonds. These gains might encourage farmers to invest in these novel sustainability tools, contributing to environmentally friendly agriculture.

## 4. Conclusions

The present study was the first to analyze the sensory properties of hidroSOStaninable almonds and consumers’ (Romania and Spain) acceptance and willingness to pay. Although the consumer panels showed similar global and attribute-specific satisfaction degrees, the trained panelists were able to establish slight but significant differences in some key attributes, with T2 almonds showing intense red color, high size, and high intensity of both sweetness and hardness attributes. The penalty analysis also showed that bitterness, which was susceptible to be improved in other treatments, was correct in T2. Consumers are now aware of the importance of the environment and the need to optimize key resources, such as water. This awareness may explain consumers’ willingness to pay a higher price for hydroSOS almonds, which will lead to higher incomes and benefits for farmers. These results lead us to conclude that controlling stress in almond trees with deficit irrigation strategies can increase water productivity and farmers’ profits from producing of environmentally friendly products without significantly changing the sensory profile and the consumers’ satisfaction. 

## Figures and Tables

**Table 1:** Sensory attributes, reference materials, and their corresponding intensities, used for the descriptive analysis of almonds.

Descriptor	Definition	Reference ^‡^	Intensity
**Appearance**			
Color	The intensity of color from light to dark	L* = 51.3; a* = 20.6; b* = 38.8	1.0
		L* = 51.3; a* = 20.6; b* = 38.8	5.0
		L* = 51.3; a* = 20.6; b* = 38.8	10.0
Size	The visual width of the of the almond from side to side	8–9 mm	1.0
		13–14 mm	5.0
		17–18 mm	9.0
Roughness	The number of hills and valleys perceived by the human eye on the almond surface (visual measured)	0%	1.0
		50%	5.0
		100%	10.0
**Basic Taste and Flavor**			
Saltiness	The basic taste associated with a sodium chloride solution	0.15% NaCl	1.0
		0.25% NaCl	3.0
Sweetness	The basic taste associated with a sucrose solution	1% sucrose	3.0
		2% sucrose	5.0
Bitterness	The basic taste associated with a caffeine solution	0.01% caffeine	2.0
		0.02% caffeine	3.0
Astringency	A drying and puckering sensation on the mouth surface	0.03% alum	1.0
		Unripe dates	10.0
Overall nuts	Aromatics related to nuts in general	Mix of grinded Hacendado Nutget:hazelnut, 1:1	5.5
Almond ID	Aromatics reminiscent of almond	Marcona almonds	6.5
Benzaldehyde like	Artificial almond or cherry aromatics	Aroma: almond extract Dr. Oetker	10.0
		Flavor: bitter almond	10.0
Woody	The sweet, musty, dark, and dry aromatics associated with the tree bark	Hacendado walnuts	3.0
Aftertaste	Longevity of key attributes intensity after swallow the sample	30 s	1.0
		1 min	3.0
		1.5 min	6.0
**Texture**			
Hardness	The force required to bite completely through the sample with molar teeth. Evaluated on the first bite down with the molars	Baby Bell light cheese	3.0
		Sugus chewy candy	6.0
		Hacendado almond	7.5
		Solano candy	10.0
Cohesiveness	The degree to which the sample deforms prior to breaking apart when compressed between molars	Hochland cheese slices	3.5
		Hacendado raisins	6.5
		Sugus chewy candy	8.0
Crispiness	The intensity of audible noise at first chew with molars	Nestlé cheerios	5.5
		Nestlé fitness	7.0
Fracturability	The force needed to break the almond. The evaluation was done with the molars after first chew	Nestlé cheerios	2.5
		Nestlé fitness	5.0
Adhesiveness	The effort needed to completely remove the sample from the teeth; measured after 5 chews	Kraft Miracle whip light dressing	4.5
		Marshmallow fluff	6.5
		Jif creamy peanut butter	8.5

^‡^ Intensities are based on a 10-point numerical scale with 0.5 increments, where 0 means “none” and 10 means “extremely strong”.

**Table 2:** Descriptive sensory analysis of raw almonds as affected by deficit irrigation. Scale used ranged from 0 = no intensity to 10 = extremely strong intensity.

Irrigation Treatments	Color	Size	Roughness	Saltiness	Sweetness	Bitterness	Astringency	Overall Nuts	Almonds ID	Benzaldehyde-like	Woody	Aftertaste	Hardness	Cohesiveness	Crispiness	Fracturability	Adhesiveness
**ANOVA Test ^†^**																	
	***	*	***	NS	*	NS	NS	NS	NS	NS	NS	NS	***	NS	NS	NS	NS
**Tukey Multiple Range Test ^‡^**																	
T1	4.0 c	8.0 a	5.3 b	0.5	3.3 ab	0.6	0.5	5.6	5.9	0.4	1.5	5.4	4.5 b	3.0	3.3	2.1	6.7
T2	5.3 a	8.0 a	6.7 a	0.5	3.5 a	0.6	0.6	5.8	5.9	0.3	1.0	5.4	5.1 a	2.7	3.7	2.5	6.6
T3	4.2 c	7.7 b	5.3 b	0.5	3.5 a	0.6	0.6	5.9	6.2	0.3	1.8	6.1	5.6 a	3.3	4.0	2.1	6.5
T4	4.7 b	7.6 b	6.2 a	0.4	2.7 b	0.5	0.7	5.4	5.9	0.3	1.9	6.1	5.6 a	3.3	4.2	2.5	6.4

^†^ NS = not significant at *p* < 0.05; *, **, and *** significant at *p* < 0.05, 0.01, and 0.001, respectively. ^‡^ Values (mean of 10 trained panelists) followed by the same letter, within the same column, were not significantly different (*p* < 0.05), according to Tukey’s least significant difference test.

**Table 3:** Affective sensory analysis of raw almonds as affected by deficit irrigation and tested in two countries of Union Europe (Spain and Romania).

	Color	Size	Almond ID	Sweetness	Bitterness	Astringency	Firmness	Crispiness	Teeth Adhesion	Aftertaste	Overall
**ANOVA ^†^**											
**Irrigation**	NS	NS	NS	NS	NS	NS	NS	NS	NS	*	NS
**Country**	***	*	***	***	NS	NS	***	***	**	***	***
**Irrigation × Country**	NS	NS	***	*	NS	NS	**	***	**	***	***
**Tukey Multiple Range Test ^‡^**											
**Irrigation**											
**T1**	7.0	7.0	6.7	6.6	6.4	6.5	6.7	6.9	6.3	6.5 ab	6.6
**T2**	7.0	7.2	6.8	6.6	6.5	6.5	6.5	6.9	6.2	6.2 b	6.5
**T3**	7.2	7.1	7.0	6.7	6.5	6.6	6.9	7.1	6.5	6.6 ab	6.9
**T4**	7.0	7.2	7.0	6.8	6.4	6.5	6.7	7.0	6.7	6.8 a	7.0
**Country**											
**Spain**	6.8 b	6.9 b	6.3 b	6.3 b	6.5	6.6	6.3 b	6.4 b	6.1 b	6.0 b	6.3 b
**Romania**	7.2 a	7.3 a	7.1 a	6.9 a	6.5	6.5	6.9 a	7.3 a	6.6 a	6.7 a	7.0 a
**Irrigation × Country**											
**Spain**											
**T1**	6.7	6.9	6.2 b	6.2 c	6.6	6.6	6.2 ab	6.3 d	6.2 ab	6.1 bc	6.2 bc
**T2**	6.6	6.8	6.1 b	6.2 c	6.4	6.3	6.0 b	6.3 cd	5.9 b	5.6 c	6.0 c
**T3**	7.0	7.2	6.4 ab	6.4 b	6.4	6.8	6.5 ab	6.6 abcd	6.2 ab	6.2 abc	6.5 abc
**T4**	6.8	6.9	6.5 ab	6.3 bc	6.4	6.6	6.4 ab	6.4 bcd	6.3 ab	6.3 abc	6.4 bc
**Romania**											
**T1**	7.2	7.1	7.0 ab	6.8 ab	6.3	6.5	6.9 ab	7.3 ab	6.4 ab	6.7 ab	6.8 abc
**T2**	7.1	7.5	7.1 a	6.7 ab	6.5	6.6	6.7 ab	7.2 abc	6.4 ab	6.4 abc	6.8 abc
**T3**	7.3	7.1	7.2 a	6.8 ab	6.5	6.5	7.1 a	7.4 a	6.7 ab	6.8 ab	7.0 ab
**T4**	7.1	7.4	7.3 a	7.0 a	6.5	6.5	6.9 ab	7.4 a	6.9 a	7.1 a	7.3 a

^†^ NS = not significant at *p* < 0.05; *, **, and *** significant at *p* < 0.05, 0.01, and 0.001, respectively. ^‡^ Values (mean of 100 consumers) followed by the same letter, within the same column, were not significantly different (*p* < 0.05), according to Tukey’s least significant difference test.
